# Echocardiographic Normal Reference Ranges for Non-invasive Myocardial Work Parameters in Pediatric Age: Results From an International Multi-Center Study

**DOI:** 10.3389/fcvm.2022.792622

**Published:** 2022-04-25

**Authors:** Jolanda Sabatino, Isabella Leo, Antonio Strangio, Sabrina La Bella, Nunzia Borrelli, Martina Avesani, Manjit Josen, Josefa Paredes, Enrico Piccinelli, Domenico Sirico, Valeria Pergola, Alain Fraisse, Salvatore De Rosa, Ciro Indolfi, Giovanni Di Salvo

**Affiliations:** ^1^Division of Paediatric Cardiology, Department of Women's and Children's Health, University Hospital Padua, Padua, Italy; ^2^Pediatric Research Institute (IRP) “Città della Speranza”, Padua, Italy; ^3^Division of Cardiology, Department of Medical and Surgical Science, Magna Graecia University, Catanzaro, Italy; ^4^Cardiovascular Research Center, Magna Graecia University, Catanzaro, Italy; ^5^Department of Paediatric Cardiology, Royal Brompton Hospital, London, United Kingdom; ^6^National Heart and Lung Institute, Imperial College, London, United Kingdom; ^7^Department of Cardiac Thoracic Vascular Sciences and Public Health, University of Padua, Padua, Italy

**Keywords:** myocardial work indices, advanced echocardiography, speckle tracking analysis, systolic function, congenital heart disease

## Abstract

**Aims:**

This international multi-center study aimed to demonstrate the feasibility and reliability of non-invasive myocardial work (MW) parameters in the pediatric population, and to provide normal reference ranges for this useful echocardiographic tool in this specific subset of patients.

**Methods and Results:**

In this retrospective multi-center study involving three pediatric laboratories, 150 healthy children and adolescents (mean age of 10.6 ± 4.5, 91 males) were enrolled. A complete echocardiographic examination has been performed, including global longitudinal strain (GLS) assessment. The following parameters of non-invasive MW have been obtained through a dedicated software: global work index (GWI), global constructive work (GCW), global work waste (GWW), and global work efficiency (GWE), using left ventricular (LV) strain loops and non-invasive brachial artery cuff pressure values. The lowest expected values were for GWI 1,723 mmHg% in males and 1,682 mmHg% in females, for GCW 2,089 and 2,106 mmHg%, for GWE 95.9 and 95.5% whereas the highest expected value for GWW was 78 mmHg% in men and 90 mmHg% in women. The univariable and multivariable analysis showed significant associations between either GWI or GCW with SBP (β coefficient = 0.446, *p* < 0.001; β coefficient = 0.456, *p* < 0.001, respectively) and LV GLS (β coefficient = −0.268, *p* = 0.001; β coefficient = −0.233, *p* = 0.003, respectively). Inter- and intra-observer variability showed good reproducibility of non-invasive MW parameters.

**Conclusion:**

Non-invasive MW parameters were feasible and reliable in the pediatric population. This study provided normal reference ranges of these useful echocardiographic indices.

## Introduction

The evaluation of left ventricular (LV) global and regional function is a crucial step of the pediatric echocardiographic examination, providing important diagnostic and prognostic information in several clinical contexts (1, 2).

Myocardial deformation analysis sheds new light on the study of myocardial mechanics in the last decade (3–5). The two-dimensional (2D) speckle-tracking echocardiography (STE) has been universally recognized as a reliable technique, when compared with traditional parameters of LV systolic function. Its capability to assess not only longitudinal, but also radial, circumferential, and torsional deformation, adds pivotal information on cardiac performance in the pediatric population (3–6).

On the other hand, the relative load dependency of LV strain makes myocardial deformation indices scarcely accurate in specific clinical settings (7).

Invasively measured myocardial work (MW) parameters have been employed to estimate ventricular contractility since the 1970s (8–10). MW reflects the myocardial stroke work and the consumption of oxygen; it has been historically calculated as the area of the invasively obtained LV pressure-volume loop, thus limiting its routine use in clinical practice.

More recently, Russell et al. (11) proposed a novel non-invasive method for calculating MW, on the basis of speckle-tracking analysis with the estimation of LV pressure from brachial artery cuff pressure.

In this regard, accounting for multiple hemodynamic factors, MW indices have been shown to provide a more accurate estimation of systolic function compared with the strain measurements alone (12).

The Normal Reference Ranges for Echocardiography (NORRE) sub-study demonstrated an optimal reproducibility, presenting reference ranges for non-invasive MW in adult patients. (13). However, no data on reference ranges of MW indices have been reported up to now in pediatric age.

Therefore, the present study aimed (i) to establish normal reference limits for MW parameters in healthy children and adolescents and (ii) to evaluate the impact of age, gender, and body surface area (BSA) on normal reference ranges.

## Methods

### Study Population

In this retrospective multi-center study, 150 healthy children and adolescents were included. Those subjects were identified from those who underwent complete echocardiograms for clinical indications (heart murmur, atypical chest pain, syncope, and family history of congenital heart diseases) from databases of the following institutions: (1) Pediatric Echocardiographic Laboratory of the Magna Graecia University Hospital of Catanzaro (Italy); (2) Pediatric Echocardiographic Laboratory of the Royal Brompton Hospital of London (United Kingdom); (3) Pediatric Echocardiographic Laboratory of the Department of Women's and Children University Hospital of Padua (Italy). Retrospective use of clinical data for this study was approved by the local competent clinical director in charge.

Demographic data such as gender, age, weight, height, and BSA have been collected for each subject. Moreover, non-invasive blood pressure values have been measured for every individual at the time of echocardiographic examination.

Inclusion criteria were: age between 0.5 and 18 years and normal cardiac structure and function at cardiovascular examination. Children were selected among those referred to our ambulatory for different clinical indications (heart murmur, chest pain of unknown origin, or as a volunteer among relatives of patients with simple congenital heart disease). All the selected subjects have an electrocardiogram and a complete echocardiography examination excluding any cardiovascular abnormalities. Exclusion criteria were: poor acoustic window, poor compliance, marked sinus arrhythmia or frequent extra-systole, any cardiac and extracardiac abnormality, and the presence of any genetic syndrome or disease.

#### Echocardiographic Examination

Echocardiographic assessment has been performed using a GE E95 ultrasound system (GE Vivid E95; Horten, Norway). Both M5S-D and 6SD transducers (GE Healthcare) have been used, according to the age and body size of the patient. A complete echocardiographic examination has been performed as previously described (11–13). Moreover, 2D 4-chambers, 3-chambers, and 2-chambers apical views were acquired with a frame rate of 60–100 frames/s. The acquired images were subsequently transferred to an offline workstation (Echopac V.202, GE Healthcare) to perform strain analysis using a dedicated software. In order to calculate global and regional 2D STE myocardial longitudinal strain, the endocardial border was manually traced, and the region of interest (ROI) automatically created by the software was then adjusted. Once strain analysis curves were obtained, a dedicated function of the GE software was used for MW estimation. The non-invasive blood pressure systolic and diastolic values, obtained by a brachial-cuff aneroid sphygmomanometer, were plugged into the software. The time of open and closure of the aortic and mitral valve was identified by the operator on the basis of the 3-chamber recording, as requested for the synchronization of strain and pressure data. Thus, the global myocardial work index (GWI) has been calculated as previously described, along with derived parameters:

- Global myocardial constructive work (GCW): work performed by a segment during shortening in systole plus negative work during lengthening in Isovolumic Relaxation (IVR);
- Global myocardial wasted work (GWW): negative work performed by a segment during lengthening in systole plus work performed during shortening in IVR;
- Global myocardial work efficiency (GWE): constructive work divided by the sum of constructive and wasted work (0–100%).

#### Data Analysis and Statistics

Data distribution was assessed by visual inspection of frequency histograms. The Kolmogorov–Smirnov test was used to test for normality. Continuous variables are presented as mean ± standard deviation (*SD*) for normal distribution or as median (interquartile range) for non-normal distribution. Categorical data are expressed as percentages. Comparison between study groups was analyzed using the unpaired *t*-test or the Mann–Whitney U test for normally and non-normally distributed continuous variables, respectively.

Pearson's correlation was used to assess correlations between variables. Univariable and multivariable linear regression analyses were calculated to evaluate the association between MW indices and demographic or echocardiographic parameters. Inter and intra-rater consistency was assessed in a sample of patients (*n* = 20) using the Bland–Altman analysis (14) and the intraclass correlation coefficients.

For sample size calculation, we estimated the expected data distribution of GWI from previously published data involving adults and adolescents. We calculated a minimum sample size of 65 individuals to reach a power of 95%. However, the total number of subjects was increased to warrant a minimum number per age group.

All statistical analyses were performed using SPSS v.21 (SPSS Inc., Chicago, IL, USA). A two-tailed *p*-value of 0.05 was considered significant.

## Results

### Baseline Demographic and Clinical Data

Table 1 summarizes the main characteristics of the study population. A total of one hundred fifty children and adolescents were included with an overall mean age of 10.6 ± 4.5 (age range 0.5–17.9 years). Of those, 91 were males (mean age 11.3 ± 4.4, age range 0.7–17.9 years) and 59 were females (mean age 9.5 ± 4.5 years, age range 0.5–16.6 years). Seven patients (4.6%) were 6-to-12-month-old, 16 (10.6%) patients were 1-to-5-year-old, 33 (22%) were 6-to-9-year-old, 39 (26%) were 10-to-13-year-old, and 55 (36%) were 14-to-18-year-old. Significant differences in height (*p* = 0.002), weight (*p* = 0.003), BSA (*p* = 0.001), diastolic blood pressure (*p* = 0.040), and LV end-diastolic diameter (LVEDD) (*p* = 0.020) between male and female subjects were observed. There were no significant differences in LV ejection fraction (EF) and LV global longitudinal strain (GLS) between sexes.

**Table 1:** Characteristics of the population.

**Parameters**	**Total (*n =* 150)**	**Male (*n =* 91)**	**Female (*n =* 59)**	**p-Value**
Age (years)	10.6 ± 4.5	11.3 ± 4.4	9.5 ± 4.5	0.014
Age range (years)	0.5–17.9	0.7–17.9	0.5–16.6	
Height (cm)	142.6 ± 26.9	148.26 ± 26	134 ± 26	0.002
Weight (kg)	41.5 ± 19	45.3 ± 19.7	35.5 ± 16.3	0.003
Body surface area (m^2^)	1.27 ± 0.4	1.35 ± 0.4	1.13 ± 0.35	0.001
Systolic blood pressure (mmHg)	112 ± 9.4	113 ± 9.7	110.3 ± 8.6	0.072
Diastolic blood pressure (mmHg)	69 ± 7.6	70 ± 7.6	67 ± 7.3	0.013
Ejection fraction (%)	64 ± 4.9	64.6 ± 5.3	64.2 ± 4.3	0.65
GLS (%)	−21 ± 2.5	−20.9 ± 1.7	−21.1 ± 3.4	0.55
LVEDD	39 ± 7	41 ± 7.2	38 ± 6.4	0.02
LVEDD Zscore	−0.35 ± 1.03	−0.4 ± 0.94	−0.3 ± 1.16	0.55
LVESD	25.4 ± 4.9	26 ± 5	24.6 ± 4.8	0.08
LVESD Zscore	0.01 ± 1.07	−0.06 ± 0.93	0.07 ± 1.25	0.50

*GLS, Global longitudinal strain; LVEDD, Left ventricular end diastolic diameter; LVESD, Left ventricular end systolic diameter*.

### Global Myocardial Work Parameters

**Figure 1:**
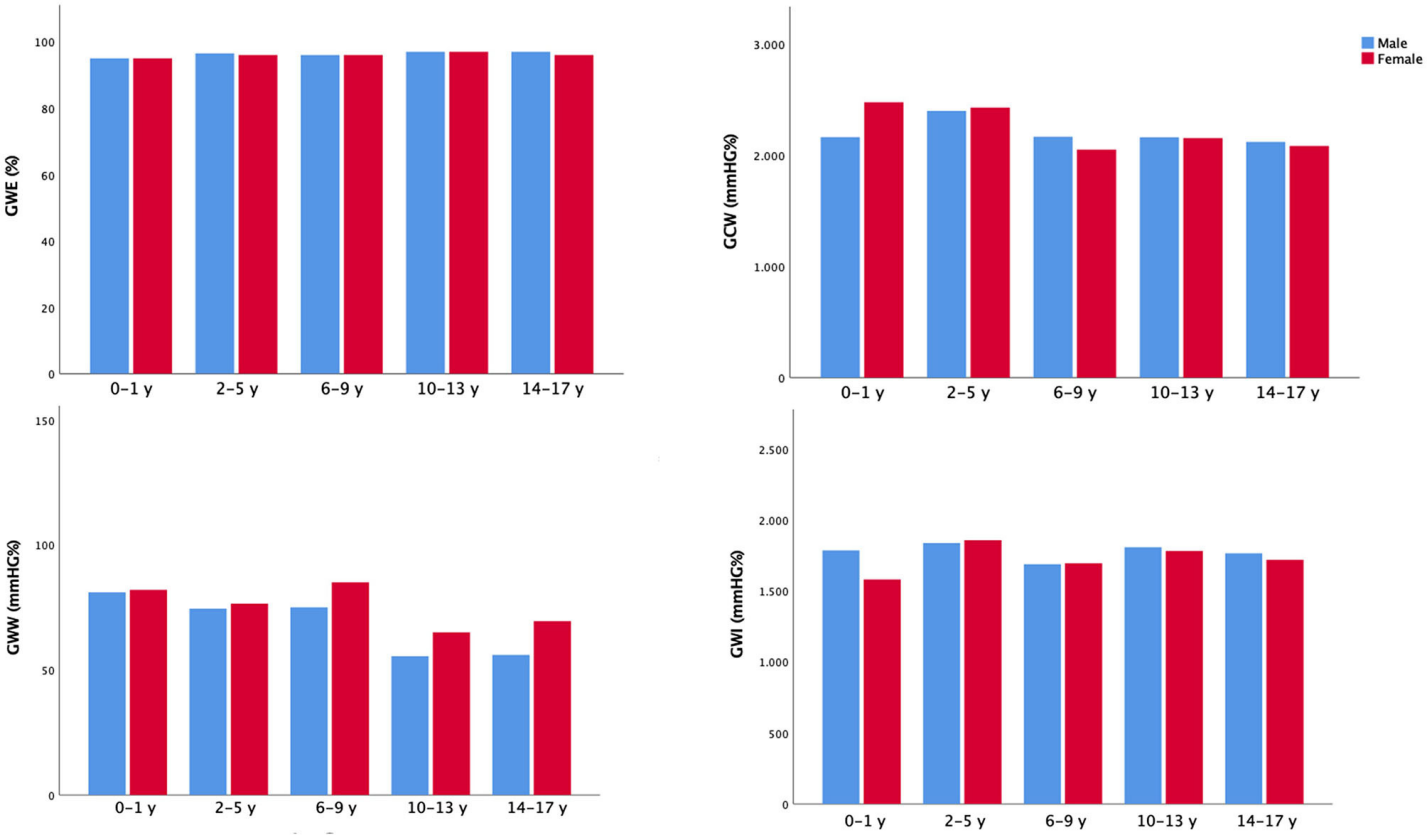
Non-invasive myocardial work indices in the study population. Mean values of non-invasive myocardial work parameters according to gender and age categories.

Two-dimensional MW parameters acquired from the study population are shown in Table 2 and in Figure 1. The lowest expected values were 1,723 mmHg% in males and 1,682 mmHg% in females for GWI, 2,089 mmHg% and 2,106 mmHg% for GCW, and 95.9 and 95.5% for GWE, respectively. The highest expected value for GWW was 78 mmHg% in men and 90 mmHg% in women. GWI, GCW, and GWE were slightly lower in females than in males, while the opposite occurred for GWW, with no statistical significance.

**Table 2:** Myocardial work non-invasive parameters.

**Parameters**	**Total, mean ±*SD* or median (IQR)**	**Total, 95% *CI***	**Male, mean ±*SD* or median (IQR)**	**Male, 95% *CI***	**Female, mean ±SD or median (IQR)**	**Female, 95% *CI***	***p*-Value**
GWI, mmHG%	1,760 ± 228	1,723–1,796	1,767 ± 212	1,723–1,811	1,748 ± 252	1,682–1,814	0.62
GCW, mmHG%	2,141 (306)	2,115–2,204	2,159 (338)	2,089–2,201	2,111 (294)	2,106–2,259	0.42
GWW, mmHG%	69 (46)	68–80	63 (39)	63–78	71 (48)	70–90	0.13
GWE, %	96 (2)	95.9–96.3	96 (2)	95.9–96.5	96 (2)	95.5–96.3	0.17

*GWI, Global work index; GCW, global constructive work; GWW, global wasted work; GWE, global work efficiency*.

There were significant linear correlations between GWI and GCW with both systolic blood pressure (SBP) and GLS (*p* < 0.001 for all). No significant correlations were observed between GWE and GWW with SBP and between GWW and GLS (*p* = NS); there was a significant correlation between GWE and GLS (*p* = 0.020).

As to demographic variables, GCW, GWW, and GWE correlated significantly with age (*R* = −0.267, *p* < 0.001, *R* = −0.203, *p* = 0.012, and *R* = 0.162, *p* = 0.047, respectively); GCW and GWW with BSA (*R* = −0.289, *p* = 0.001, and *R* = −0.178, *p* = 0.035, respectively).

On both univariable and multivariable analyses, we observed significant associations between either GWI or GCW with SBP (β coefficient = 0.446, *p* < 0.001; β coefficient = 0.456, *p* < 0.001, respectively) and LV GLS (β coefficient = −0.268, *p* = 0.001; β coefficient = −0.233, *p* = 0.003, respectively).

No associations were found at multivariable analysis between any of the MW parameters and age, sex, or BSA.

### Repeatability and Reproducibility

**Figure 2:**
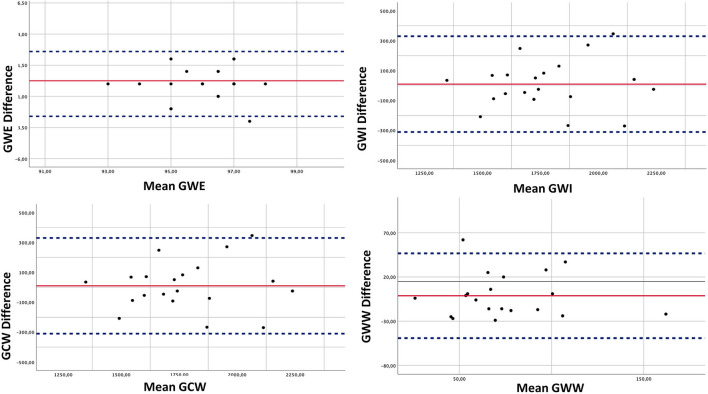
Inter-observer reproducibility. The Bland Altman analysis was performed to assess in a sample of patients the inter-observer variability of the main indices of non-invasive myocardial work. Continuous line represents mean values for each parameter, and dotted lines represent bias and 95% limits of agreement.

**Figure 3:**
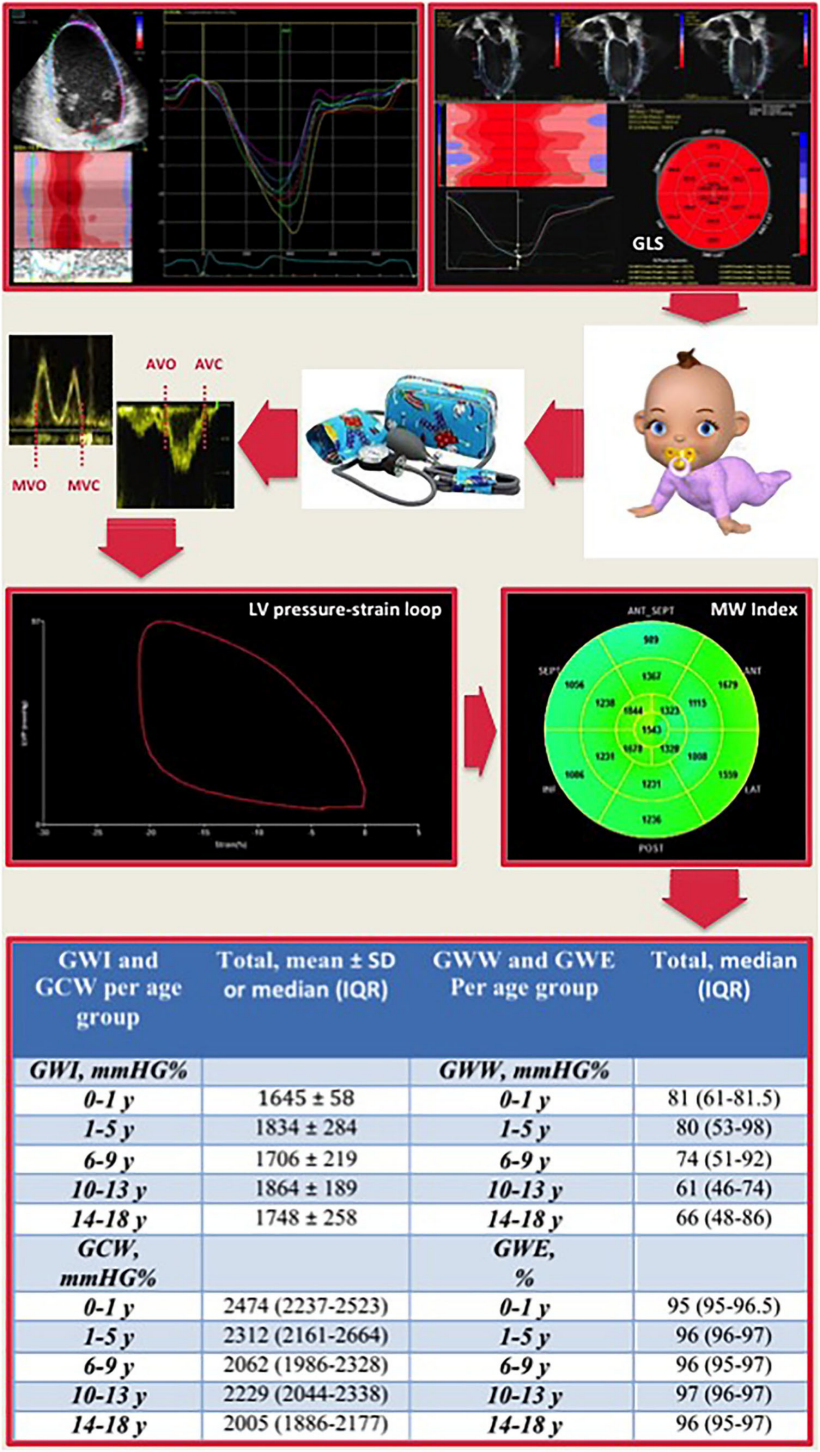
Representative schema of myocardial work calculation with reference cut-off values for age subgroups. LV global longitudinal strain measurement combined with the non-invasive blood pressure systolic and diastolic values, obtained by a brachial-cuff aneroid sphygmomanometer, is plugged into dedicated software. The time of opening and closure of the aortic and mitral valve is identified by the operator. Then, a trace showing the LV pressure-strain loop and a 17-segment bull's-eye of the MW index is calculated. At the bottom, the table resumes the normal reference ranges for each myocardial work parameter in the overall population and for each age group. GLS, global longitudinal strain; AVC, aortic valve closure; AVO, aortic valve opening; MVC, mitral valve closure; MWO, mitral valve opening; LV, left ventricular; MW, myocardial work.

Intra-observer and inter-observer indices of variability for MW parameters are shown in Table 3. Intra-observer and inter-observer analyses demonstrated good repeatability and reproducibility of MW parameters (Table 3; Figure 2). The Bland-Altman plot showed a low bias with narrow limits of agreement and no evidence of relevant heteroscedasticity for all MW indices assessed (Figure 2). Figure 3 reports a practical example of calculation of non-invasive Myocardial Work, along with summary normative results.

**Table 3:** Intra- and inter-observer reproducibility of myocardial work parameters.

**Parameters**	**Intraclass**	**95% confidence**	***p*-Value**
	**coefficients (ICCs)**	**interval**	
**Inter-observer variability**			
GWI	0.886	0.711–0.955	<0.001
GCW	0.761	0.413–0.904	0.001
GWW	0.839	0.588–0.936	<0.001
GWE	0.722	0.281–0.891	0.005
**Intra-observer variability**			
GWI	0.923	0.708–0.980	<0.001
GCW	0.803	0.280 −0.950	0.010
GWW	0.842	0.368–0.961	0.007
GWE	0.899	0.614–0.975	0.001

*GWI, Global work index; GCW, global constructive work; GWW, global wasted work; GWE, global work efficiency*.

## Discussion

This international multi-center retrospective study is the first to provide normal reference ranges for MW parameters in healthy children and adolescents. We were also able to demonstrate good reproducibility of this non-invasive method for assessing MW in a population of children and adolescents.

Our result, moreover, confirms that MW parameters are not influenced by age, sex, or BSA in children, as previously demonstrated in healthy adults and adolescents (13, 15).

On the contrary, both GWI and GCW are influenced by SBP and LV GLS at univariable and multivariable analyses, in agreement with data reported in studies on normal adults and adolescents (13, 15).

This study, therefore, confirms the absence of a strong dependence of MW on age, gender, and BSA, by highlighting the association between GWI and GCW with SBP and LV GLS. Furthermore, SBP has been shown to increase with age (16), whereas LV GLS decreases slightly with age (17). This reverse trend between the two parameters, which are both input variables for determining MW indices, could explain, at least in part, the relative not-surprising independence of MW parameters from age. However, MW considers deformation along with afterload, providing additional information to strain in assessing cardiac performance.

### Myocardial Work and Ventricular Function

It is well known that longitudinal deformation parameters are influenced by changes in loading conditions, which may limit their accuracy (7). For example, it has been previously demonstrated that an increase in afterload may lead to a systolic impairment in presence of preserved or even increased MW (7).

Myocardial work has been measured invasively and used as a marker of ventricular contractility for decades (8–10). Russell et al. have introduced a novel method for calculating non-invasive MW (11, 12), which takes into consideration speckle-tracking analysis and LV pressure estimated from brachial artery cuff pressure and good reproducibility (13).

Myocardial work parameters have been shown to be reliable predictors of response to cardiac resynchronization therapy (CRT) in patients with heart failure with reduced ejection fraction (18). Chan et al. have demonstrated that MW indices are significantly increased in patients with hypertension compared to controls, regardless of normal GLS values (19).

Most recently, a previous study from our group demonstrated MW indices as being sensitive and early indicators of myocardial ischemia during transient acute coronary occlusion (20).

### Myocardial Work Parameters in Pediatric Age

Our study sought to provide norms for future studies investigating the clinical utility of MW parameters in children and adolescents, especially in those with congenital or acquired cardiac disease.

Non-invasive MW parameters have already shown insightful diagnostic yield in two studies conducted on pediatric population. Children with Turner syndrome demonstrated increased MW compared to age-matched controls, as shown by Oberhoffer et al. (21). Results from this study may relate to the early development of arterial stiffening widely reported in such patients.

A recent study from our group evaluated the diagnostic performance of non-invasive MW indices in a cohort of children and adolescents with Kawasaki disease (KD) with and without coronary dilatation (12). It was thus demonstrated that non-invasive MW indices are reduced in children with KD, even in the absence of coronary aneurysm, and that they can detect subtle myocardial abnormalities better than GLS and LV EF.

The feasibility of non-invasive MW in our healthy pediatric population was optimal, as 2D imaging quality is usually greater in pediatrics than adults. Thus, we had to exclude no subject from this study because of poor image quality. On the other side, intra- and inter-observer variability for MW indices were relatively higher than the previous rates published in adults (22). This can be partially explained by the fact that we did not perform any specific acquisition for valvular timings, and this may be particularly relevant in pediatric patients in the presence of a relatively high heart rate. Moreover, measurement of non-invasive blood pressure in children could be challenging, especially in pre-school children or newborns.

### Limitations

This is a retrospective study. Thus, our findings should be further validated in large prospective studies.

Measurement of non-invasive MW, in this study, is derived from speckle-tracking analysis, carrying with itself the same well-known limitations of the speckle-tracking techniques. Indeed, while acoustic windows are usually more echogenic in younger patients, poor image quality can be related to the lack of children's compliance, the relatively high heart rate, and lung artifacts.

Age distribution of patients may have influenced statistical analysis. In particular, since we did not set up a fixed number of individuals per age class to minimize selection biases, different age classes do not have the same size. For the same reason, though we do report data for all the different age classes, these should be considered as informative explorative data, as some age subgroups included a limited number of individuals and their relative normative data may not be sufficiently robust.

Our results do not apply to subjects from 0.0 to 0.5 years of age. In fact, these were not included in this study because they represent a different physiological background. In particular, relevant maturational changes in myocardial deformations compared to older children are to be expected. For this reason, the 0–0.5 years age groups merit a dedicated study to find normal values.

Finally, the non-invasive MW evaluation currently can only be performed by means of General Electric echocardiography machines and EchoPAC software. This can reduce its broad utilization; moreover, we might speculate vendor-specific differences in GMW-parameters when other manufacturers will upgrade their software with these functionalities. Would other vendors develop their MW indices? This would most probably need a dedicated normative study.

## Conclusions

This study provides applicable 2D normative reference ranges for MW parameters. Interestingly, we found that MW parameters are not substantially influenced by age, gender, and BSA. Norms established by our study, thus, provide an operational framework for future studies searching on MW parameters for clinical applications in children and adolescents with congenital or acquired cardiac disease.

## Data Availability Statement

The raw data supporting the conclusions of this article will be made available by the authors, without undue reservation.

## Ethics Statement

The studies involving human participants were reviewed and approved by Magna Graecia University. Written informed consent to participate in this study was provided by the participants' legal guardian/next of kin.

## Author Contributions

JS and GD: conceptualization. JS, IL, AS, MA, SB, NB, MJ, JP, EP, and DS: data collection and analysis. GD, VP, AF, SD, and CI: review. All authors discussed the results and contributed to the final manuscript.

## Conflict of Interest

The authors declare that the research was conducted in the absence of any commercial or financial relationships that could be construed as a potential conflict of interest.

## Publisher's Note

All claims expressed in this article are solely those of the authors and do not necessarily represent those of their affiliated organizations, or those of the publisher, the editors and the reviewers. Any product that may be evaluated in this article, or claim that may be made by its manufacturer, is not guaranteed or endorsed by the publisher.
